# Supplementary material for: Role and mechanism of NCAPD3 in promoting malignant behaviors in gastric cancer
Source: Front Pharmacol. 2024 Apr 22;15:1341039. doi: 10.3389/fphar.2024.1341039 (PMC11070777; doi:10.3389/fphar.2024.1341039)
Supplement: Supplementary file 11 [file DataSheet2.ZIP › GSEA/GO_CC/my_analysis.Gsea.1599461408869/GO_ENDOPLASMIC_RETICULUM.html]

Details for gene set GO\_ENDOPLASMIC\_RETICULUM[GSEA]

|  || Dataset | filtered\_dataset.sample\_info.cls#WT\_versus\_NCAPD3\_MUT |
| Phenotype | sample\_info.cls#WT\_versus\_NCAPD3\_MUT |
| Upregulated in class | NCAPD3\_MUT |
| GeneSet | GO\_ENDOPLASMIC\_RETICULUM |
| Enrichment Score (ES) | -0.21035253 |
| Normalized Enrichment Score (NES) | -1.6764492 |
| Nominal p-value | 0.011904762 |
| FDR q-value | 0.113571264 |
| FWER p-Value | 0.602 |
Table: GSEA Results Summary

  

Fig 1: Enrichment plot: GO\_ENDOPLASMIC\_RETICULUM      
 Profile of the Running ES Score & Positions of GeneSet Members on the Rank Ordered List

  

| SYMBOL | TITLE | RANK IN GENE LIST | RANK METRIC SCORE | RUNNING ES | CORE ENRICHMENT || 1 | 7052 | TGM2 | 17 | 1.045 | 0.0022 | No |
| 2 | 2243 | FGA | 18 | 1.039 | 0.0174 | No |
| 3 | 79803 | HPS6 | 72 | 0.853 | -0.0112 | No |
| 4 | 9489 | PGS1 | 84 | 0.820 | -0.0077 | No |
| 5 | 92140 | MTDH | 93 | 0.801 | -0.0022 | No |
| 6 | 10206 | TRIM13 | 161 | 0.716 | -0.0436 | No |
| 7 | 817 | CAMK2D | 165 | 0.710 | -0.0355 | No |
| 8 | 29940 | DSE | 174 | 0.703 | -0.0314 | No |
| 9 | 23023 | TMCC1 | 181 | 0.695 | -0.0259 | No |
| 10 | 11231 | SEC63 | 193 | 0.685 | -0.0244 | No |
| 11 | 9897 | KIAA0196 | 197 | 0.682 | -0.0167 | No |
| 12 | 3303 | HSPA1A | 206 | 0.669 | -0.0131 | No |
| 13 | 3304 | HSPA1B | 207 | 0.669 | -0.0032 | No |
| 14 | 81619 | TSPAN14 | 212 | 0.666 | 0.0034 | No |
| 15 | 126792 | B3GALT6 | 223 | 0.658 | 0.0053 | No |
| 16 | 64856 | VWA1 | 259 | 0.623 | -0.0127 | No |
| 17 | 353376 | TICAM2 | 271 | 0.617 | -0.0122 | No |
| 18 | 199857 | ALG14 | 275 | 0.615 | -0.0055 | No |
| 19 | 80124 | VCPIP1 | 282 | 0.607 | -0.0012 | No |
| 20 | 10585 | POMT1 | 284 | 0.606 | 0.0069 | No |
| 21 | 538 | ATP7A | 305 | 0.588 | 0.0000 | No |
| 22 | 2244 | FGB | 321 | 0.582 | -0.0031 | No |
| 23 | 8125 | ANP32A | 326 | 0.578 | 0.0023 | No |
| 24 | 55697 | VAC14 | 329 | 0.576 | 0.0092 | No |
| 25 | 84135 | UTP15 | 330 | 0.576 | 0.0177 | No |
| 26 | 94107 | TMEM203 | 333 | 0.574 | 0.0246 | No |
| 27 | 1203 | CLN5 | 358 | 0.562 | 0.0142 | No |
| 28 | 23184 | MESDC2 | 362 | 0.559 | 0.0201 | No |
| 29 | 10618 | TGOLN2 | 383 | 0.547 | 0.0125 | No |
| 30 | 79683 | ZDHHC14 | 397 | 0.539 | 0.0104 | No |
| 31 | 60526 | LDAH | 403 | 0.535 | 0.0143 | No |
| 32 | 147007 | TMEM199 | 410 | 0.533 | 0.0175 | No |
| 33 | 56894 | AGPAT3 | 414 | 0.527 | 0.0229 | No |
| 34 | 55326 | AGPAT5 | 421 | 0.521 | 0.0259 | No |
| 35 | 5295 | PIK3R1 | 467 | 0.493 | -0.0018 | No |
| 36 | 25923 | ATL3 | 488 | 0.480 | -0.0103 | No |
| 37 | 54920 | DUS2 | 509 | 0.470 | -0.0189 | No |
| 38 | 3433 | IFIT2 | 608 | 0.418 | -0.0888 | No |
| 39 | 10445 | MCRS1 | 617 | 0.414 | -0.0890 | No |
| 40 | 286410 | ATP11C | 637 | 0.405 | -0.0978 | No |
| 41 | 10280 | SIGMAR1 | 655 | 0.399 | -0.1051 | No |
| 42 | 94005 | PIGS | 663 | 0.395 | -0.1047 | No |
| 43 | 23250 | ATP11A | 664 | 0.395 | -0.0989 | No |
| 44 | 51283 | BFAR | 676 | 0.389 | -0.1018 | No |
| 45 | 23212 | RRS1 | 681 | 0.387 | -0.0992 | No |
| 46 | 5311 | PKD2 | 708 | 0.372 | -0.1139 | No |
| 47 | 178 | AGL | 711 | 0.368 | -0.1101 | No |
| 48 | 23640 | HSPBP1 | 735 | 0.359 | -0.1227 | No |
| 49 | 25825 | BACE2 | 756 | 0.344 | -0.1331 | No |
| 50 | 23197 | FAF2 | 760 | 0.342 | -0.1304 | No |
| 51 | 10492 | SYNCRIP | 779 | 0.334 | -0.1395 | No |
| 52 | 84522 | JAGN1 | 798 | 0.324 | -0.1487 | No |
| 53 | 57665 | RDH14 | 819 | 0.304 | -0.1598 | No |
| 54 | 26608 | TBL2 | 821 | 0.304 | -0.1561 | No |
| 55 | 1605 | DAG1 | 823 | 0.304 | -0.1524 | No |
| 56 | 1718 | DHCR24 | 832 | 0.293 | -0.1543 | No |
| 57 | 9524 | TECR | 834 | 0.292 | -0.1508 | No |
| 58 | 6732 | SRPK1 | 835 | 0.291 | -0.1465 | No |
| 59 | 1717 | DHCR7 | 836 | 0.288 | -0.1423 | No |
| 60 | 26031 | OSBPL3 | 870 | -0.281 | -0.1638 | No |
| 61 | 3309 | HSPA5 | 876 | -0.288 | -0.1634 | No |
| 62 | 27230 | SERP1 | 892 | -0.306 | -0.1706 | No |
| 63 | 51141 | INSIG2 | 903 | -0.328 | -0.1735 | No |
| 64 | 23710 | GABARAPL1 | 913 | -0.341 | -0.1755 | No |
| 65 | 51124 | IER3IP1 | 918 | -0.347 | -0.1735 | No |
| 66 | 3913 | LAMB2 | 919 | -0.348 | -0.1684 | No |
| 67 | 10232 | MSLN | 925 | -0.351 | -0.1671 | No |
| 68 | 4052 | LTBP1 | 928 | -0.353 | -0.1635 | No |
| 69 | 153830 | RNF145 | 940 | -0.363 | -0.1667 | No |
| 70 | 5329 | PLAUR | 952 | -0.373 | -0.1698 | No |
| 71 | 81544 | GDPD5 | 961 | -0.381 | -0.1704 | No |
| 72 | 4864 | NPC1 | 964 | -0.384 | -0.1663 | No |
| 73 | 493869 | GPX8 | 966 | -0.385 | -0.1614 | No |
| 74 | 5621 | PRNP | 970 | -0.387 | -0.1581 | No |
| 75 | 84919 | PPP1R15B | 979 | -0.394 | -0.1585 | No |
| 76 | 53349 | ZFYVE1 | 1018 | -0.417 | -0.1819 | No |
| 77 | 9926 | LPGAT1 | 1036 | -0.434 | -0.1887 | No |
| 78 | 7871 | SLMAP | 1038 | -0.435 | -0.1831 | No |
| 79 | 130617 | ZFAND2B | 1046 | -0.444 | -0.1820 | No |
| 80 | 80315 | CPEB4 | 1053 | -0.448 | -0.1801 | No |
| 81 | 1191 | CLU | 1056 | -0.450 | -0.1750 | No |
| 82 | 81502 | HM13 | 1057 | -0.450 | -0.1684 | No |
| 83 | 1308 | COL17A1 | 1065 | -0.455 | -0.1672 | No |
| 84 | 857 | CAV1 | 1098 | -0.475 | -0.1850 | No |
| 85 | 10098 | TSPAN5 | 1112 | -0.485 | -0.1880 | No |
| 86 | 3134 | HLA-F | 1139 | -0.506 | -0.2007 | No |
| 87 | 7444 | VRK2 | 1145 | -0.509 | -0.1972 | No |
| 88 | 831 | CAST | 1160 | -0.524 | -0.2003 | No |
| 89 | 1282 | COL4A1 | 1165 | -0.534 | -0.1956 | No |
| 90 | 665 | BNIP3L | 1185 | -0.556 | -0.2022 | Yes |
| 91 | 5154 | PDGFA | 1191 | -0.566 | -0.1978 | Yes |
| 92 | 9709 | HERPUD1 | 1202 | -0.578 | -0.1970 | Yes |
| 93 | 23657 | SLC7A11 | 1205 | -0.581 | -0.1901 | Yes |
| 94 | 4189 | DNAJB9 | 1223 | -0.598 | -0.1945 | Yes |
| 95 | 2043 | EPHA4 | 1228 | -0.602 | -0.1887 | Yes |
| 96 | 22931 | RAB18 | 1231 | -0.608 | -0.1814 | Yes |
| 97 | 54491 | FAM105A | 1241 | -0.619 | -0.1793 | Yes |
| 98 | 7057 | THBS1 | 1242 | -0.620 | -0.1702 | Yes |
| 99 | 837 | CASP4 | 1245 | -0.623 | -0.1626 | Yes |
| 100 | 154064 | RAET1L | 1276 | -0.664 | -0.1761 | Yes |
| 101 | 80328 | ULBP2 | 1277 | -0.664 | -0.1663 | Yes |
| 102 | 353091 | RAET1G | 1278 | -0.664 | -0.1566 | Yes |
| 103 | 4240 | MFGE8 | 1280 | -0.668 | -0.1475 | Yes |
| 104 | 286 | ANK1 | 1284 | -0.672 | -0.1400 | Yes |
| 105 | 253558 | LCLAT1 | 1288 | -0.679 | -0.1324 | Yes |
| 106 | 5046 | PCSK6 | 1297 | -0.699 | -0.1283 | Yes |
| 107 | 84947 | SERAC1 | 1303 | -0.707 | -0.1218 | Yes |
| 108 | 57348 | TTYH1 | 1306 | -0.710 | -0.1129 | Yes |
| 109 | 253782 | CERS6 | 1307 | -0.711 | -0.1025 | Yes |
| 110 | 195814 | SDR16C5 | 1314 | -0.728 | -0.0965 | Yes |
| 111 | 6262 | RYR2 | 1323 | -0.741 | -0.0918 | Yes |
| 112 | 84803 | GPAT3 | 1327 | -0.748 | -0.0831 | Yes |
| 113 | 7779 | SLC30A1 | 1332 | -0.755 | -0.0752 | Yes |
| 114 | 6646 | SOAT1 | 1333 | -0.755 | -0.0641 | Yes |
| 115 | 57157 | PHTF2 | 1334 | -0.755 | -0.0530 | Yes |
| 116 | 1116 | CHI3L1 | 1339 | -0.777 | -0.0447 | Yes |
| 117 | 56605 | ERO1B | 1340 | -0.778 | -0.0332 | Yes |
| 118 | 51703 | ACSL5 | 1353 | -0.800 | -0.0308 | Yes |
| 119 | 54463 | FAM134B | 1356 | -0.815 | -0.0204 | Yes |
| 120 | 64764 | CREB3L2 | 1382 | -0.930 | -0.0261 | Yes |
| 121 | 2040 | STOM | 1389 | -0.988 | -0.0163 | Yes |
| 122 | 81573 | ANKRD13C | 1393 | -1.049 | -0.0032 | Yes |
| 123 | 6782 | HSPA13 | 1394 | -1.064 | 0.0124 | Yes |
Table: GSEA details [plain text format]

  

Fig 2: GO\_ENDOPLASMIC\_RETICULUM      
 Blue-Pink O' Gram in the Space of the Analyzed GeneSet

  

Fig 3: GO\_ENDOPLASMIC\_RETICULUM: Random ES distribution      
 Gene set null distribution of ES for **GO\_ENDOPLASMIC\_RETICULUM**
